# Why Certain Repurposed Drugs Are Unlikely to Be Effective Antivirals to Treat SARS-CoV-2 Infections

**DOI:** 10.3390/v16040651

**Published:** 2024-04-22

**Authors:** Selwyn J. Hurwitz, Ramyani De, Julia C. LeCher, Jessica A. Downs-Bowen, Shu Ling Goh, Keivan Zandi, Tamara McBrayer, Franck Amblard, Dharmeshkumar Patel, James J. Kohler, Manoj Bhasin, Brian S. Dobosh, Vikas Sukhatme, Rabindra M. Tirouvanziam, Raymond F. Schinazi

**Affiliations:** 1 Center for ViroScience and Cure, Laboratory of Biochemical Pharmacology, Department of Pediatrics, Emory University School of Medicine and Children’s Healthcare of Atlanta, 1760 Haygood Drive, Atlanta, GA 30322, USA; shurwit@emory.edu (S.J.H.); ramyani.de@emory.edu (R.D.); julia.christine.lecher@emory.edu (J.C.L.); jadowns@emory.edu (J.A.D.-B.); shuling.9191@gmail.com (S.L.G.); keivanzandi@yahoo.com (K.Z.); tamara.mcbrayer@emory.edu (T.M.); famblar@emory.edu (F.A.); dharmeshkumar.j.patel@emory.edu (D.P.); jkohler35@hotmail.com (J.J.K.); 2 Center for Cystic Fibrosis & Airways Disease Research, Division of Pulmonary, Allergy & Immunology, Cystic Fibrosis and Sleep, Emory University and Children’s Healthcare of Atlanta, 2015 Uppergate Drive, Atlanta, GA 30322, USA; manoj.bhasin@emory.edu (M.B.); brian.seth.dobosh@emory.edu (B.S.D.); tirouvanziam@emory.edu (R.M.T.); 3 Morningside Center for Innovative and Affordable Medicine, Departments of Medicine and Hematology and Oncology, Emory University School of Medicine, Atlanta, GA 30322, USA; vsukhatme@emory.edu

**Keywords:** drug repurposing, SARS-CoV-2, COVID-19, coronavirus, potency, pharmacokinetics, potential efficacy, antiviral agents

## Abstract

Most repurposed drugs have proved ineffective for treating COVID-19. We evaluated median effective and toxic concentrations (EC_50_, CC_50_) of 49 drugs, mostly from previous clinical trials, in Vero cells. Ratios of reported unbound peak plasma concentrations, (C_max_)/EC_50_, were used to predict the potential in vivo efficacy. The 20 drugs with the highest ratios were retested in human Calu-3 and Caco-2 cells, and their CC_50_ was determined in an expanded panel of cell lines. Many of the 20 drugs with the highest ratios were inactive in human Calu-3 and Caco-2 cells. Antivirals effective in controlled clinical trials had unbound C_max_/EC_50_ ≥ 6.8 in Calu-3 or Caco-2 cells. EC_50_ of nucleoside analogs were cell dependent. This approach and earlier availability of more relevant cultures could have reduced the number of unwarranted clinical trials.

## 1. Introduction

Since the start of the COVID-19 pandemic, caused by severe acute respiratory syndrome coronavirus 2 (SARS-CoV-2), pharmaceutical companies and academic institutions have urgently searched for treatments and vaccines. Several specific and potent antiviral agents and vaccines have undergone various stages of clinical testing and regulatory approval. The three vaccines licensed in the USA include the Pfizer-BioNTech and Moderna COVID-19 mRNA vaccines, and the Novavax COVID-19 vaccine, which is a protein subunit vaccine [1]. They are considered safe and effective in reducing the number of individuals infected, and in decreasing the severity of the symptoms in vaccinated persons who experience breakthrough infections. However, vaccines have a limited impact for individuals with active SARS-CoV-2 infections and provide limited global protection against emerging virus strains [2,3]. Existing vaccines that target the spike protein of SARS-CoV-2 make cross resistance between vaccines theoretically possible, and the durability of protection is low, thus necessitating repeated vaccination and boosters.

Drugs developed specifically to block SARS-CoV-2 replication, as well as those considered for repurposing, are directed at viral targets other than the spike protein, making cross resistance with vaccines unlikely [4]. Remdesivir is an antiviral nucleoside analog (NA) produced by Gilead Sciences Inc which was originally developed for the treatment of hepatitis C virus (HCV), Ebola virus, and Marburg virus infections [5], but it was never approved by the Food and Drug Administration (FDA) for these indications. Intravenous (IV) remdesivir was shown to shorten the hospitalization duration of hospitalized patients with COVID-19 by 5 days (ACTT-1 trial) and improve their survival outcomes [6,7,8]. A more recent clinical study with IV remdesivir in high-risk non-hospitalized patients (PINETREE trial) demonstrated an 87% reduction in hospitalizations following a short 3-day course [9]. Remdesivir is now FDA approved for the treatment of COVID-19 in adults, and pediatric patients aged ≥ 28 days and weighing ≥ 3 kg, for both non-hospitalized patients with mild to moderate COVID-19 who are at high risk of progressing to severe disease, and in hospitalized patients [10,11]. Ritonavir-boosted nirmatrelvir (Paxlovid^TM^, Pfizer Inc New York City, NY, USA) was the first oral formulation to receive full FDA approval for home treatment in high-risk adults with co-morbidities, starting within a few days of their first symptoms [12,13,14]. Paxlovid™ was shown to reduce the risk of hospitalization or death by 89% (if administered within a few days of their first symptoms) in persons over 12 years of age [12,15]. The current FDA COVID-19 Treatment Guidelines recommend using Paxlovid for 5 days in non-hospitalized adult and pediatric patients, aged ≥12 years and weighing ≥ 40 kg, with mild to moderate COVID-19 who are at high risk of disease progression [16]. Treatment should be initiated as soon as possible and within 5 days of symptom onset [14]. Molnupiravir (Lagevrio^TM^, Merck & Co., Inc., Rahway, NJ, USA) is an orally administered antiviral NA which received emergency use approval from the FDA for home treatment in high-risk adults with co-morbidities, starting within a few days of their first symptoms [17]. Molnupiravir is a 5′-ester prodrug of *N*^4^-hydroxycytidine, which was originally discovered as a broad-spectrum antiviral agent in our laboratories at Emory University and Pharmasset Inc., and was routinely used as a positive control in antiviral screening assays, including for HCV and other viruses [18,19,20,21]. It was repurposed as an ester prodrug by Drug Innovation Ventures at Emory (DRIVE LLC, Atlanta, GA, USA) for coronaviruses, and licensed to Ridgeback Biotherapeutic LP, and then to Merck & Co., Inc. [22]. Molnupiravir, like most antiviral NAs, does not require boosting and is less susceptible to drug–drug interactions (DDIs) than protease inhibitors. The drug’s mechanism of action involves inducing lethal mutations in the SARS-CoV-2 genome. Furthermore, molnupiravir can have mutagenic effects in mammalian cells and is not recommended in pregnant women [23,24,25,26]. Obeldesivir (GS-5245, Gilead Sciences Inc, San Francisco, CA, USA) is an orally administered 5′-isobutyl prodrug of GS-441524 (parent compound of remdesivir) being evaluated in two Phase 3 clinical trials (ClinicalTrials.gov Identifiers: NCT05603143 and NCT05715528) [27,28].

The drug-resistant lineages of SARS-CoV-2 have been isolated in infected cells serially passaged in increasing concentrations of remdesivir (or GS-441524) and nirmatrelvir [29,30]. Resistance to remdesivir (or GS-441524) is associated with mutations in the RNA-dependent RNA polymerase gene, resulting in a 2.5 to 10-fold increase in EC_50_ [29,31], while resistance to nirmatrelvir is associated with mutations in the 3-chymotrypsin-like cysteine protease (3CL^pro^) gene [30]. Documented clinical cases of resistance to remdesivir (or GS-441524) and nirmatrelvir are rare [32,33], likely due to the relatively short duration of treatment of most patients with COVID-19. The most common 3CL^pro^ mutations selected for nirmatrelvir in the clinic, one year after the FDA approval of nirmatrelvir–ritonavir, were E166V and S144E, which reduce the inhibitory activity of nirmatrelvir by >100-fold and were present in <1 per million sequences [33]. Molnupiravir also has a high barrier to resistance in vitro [34]. However, molnupiravir’s signature mutations were detected in a study of newly infected persons in close contact with molnupiravir-treated individuals who had not cleared the virus [35]. Since resistance to these antivirals may occur, it is important to monitor for any increase in resistant SARS-CoV-2 strains in the population, especially with the emergence of new SARS-CoV-2 variants. Since drug resistance may occur, and mutant SARS-CoV-2 lineages continue to emerge, further drug development is needed to expand treatment options.

Antiviral agents developed for other diseases (e.g., HIV, viral hepatitis, and for veterinary viruses) have also been considered as potential agents to use against SARS-CoV-2, but none have been useful when rigorously evaluated in cell culture or in clinical trials [36,37,38,39,40,41]. Other medications approved for unrelated conditions (e.g., antimalarial drugs [42]; COX inhibitors [43,44]; glucocorticoids [45]; non-steroidal anti-inflammatory agents [46,47,48]; immunosuppressive monoclonal antibodies [49]; histamine blockers (H1 or H2 receptors) [50,51]; leukotriene inhibitors [52]; antioxidants [53,54]; mucolytics [55,56]; antibacterial antibiotics [57,58]; anti-fungal compounds [59]; anti-parasitic agents [60,61]; lipid lowering drugs [62]; oncology drugs [63,64]; selective serotonin uptake inhibitor antidepressants [65]; drugs used to treat diabetes [66]; lipid lowering agents [67]; dietary supplements [68,69]; and food additives [70]) have all been considered. However, most of these repurposed drugs have shown marginal or no benefit in clinical studies (discussed below).

In this study, we provide a side-by-side evaluation of the antiviral activity of 49 drugs previously considered for inclusion in regimens for treating individuals infected with SARS-CoV-2, using remdesivir^TM^ as a positive control in a cell-based assay system. The in vitro potency measurements of all these drugs were compared with peak drug concentrations observed in the clinic to assess the potential clinical relevance of these compounds [71].

## 2. Materials and Methods

### 2.1. Drug Candidates

All drug candidates were purchased from Sigma-Aldrich (St. Louis, MO, USA) or a reliable source (e.g., extraction from prescription drugs) with at least 98% purity as determined by LC-MS-MS.

### 2.2. Cells and Viruses

Cell and virus culture techniques were adapted from previous projects in our laboratory [72,73]. Briefly: An African green monkey kidney epithelial cell line (Vero-CCL-81; CCL-81) [74], a human colorectal adenocarcinoma cell line (Caco-2; HTB-37™), a human lung cancer cell line (Calu-3; HTB-55)s [75,76], and a T-lymphoblastoid cell line (CCRF-CEM; CCL-119) were all obtained from the American Type Culture Collection (ATCC, Manassas, VA, USA). A hepatic cancer cell line (Huh-7; JCRB0403) was obtained from the Japanese Collection of Research Bioresources Cell Bank (JCRB, Osaka, Japan) [77]. Primary human peripheral blood mononuclear cells (PBMs) were isolated from blood pooled from multiple donors purchased from the New York Blood Center (NYBC; New York City, NY, USA) and stimulated with phytohemagglutinin [78]. Vero cells were cultured in minimum essential medium (MEM) supplemented with 10% fetal bovine serum (FBS). Huh-7 cells were cultured in Dulbecco’s modified eagle medium (DMEM) with 10% FBS. Caco-2 and Calu-3 cells were cultured in DMEM/F12 supplemented with 10% FBS. CEM and PBM cells were cultured in Roswell Park Memorial Institute (RPMI) 1640 Medium supplemented with 10 and 20% FBS, respectively. Media was supplemented with 2 µM L-glutamine and 100U Penicillin/Streptomycin. Media was purchased from Corning (Corning, NY, USA), and a single lot of heat-inactivated FBS (VWR, Radnor, PA, USA) was used. SARS-CoV-2, isolate hCoV-19/USA-WA1/2020, NR-52281, deposited by the Centers for Disease Control and Prevention and obtained through BEI Resources, NIAID, NIH: SARS-Related Coronavirus 2, was used.

### 2.3. In Vitro Evaluation of Compounds versus SARS-CoV-2

The anti-SARS-CoV-2 EC_50_ values and cytotoxicities (CC_50_) of drugs were initially evaluated in Vero cells. Drugs having the following properties were subsequently evaluated in human cells: (1) unbound C_max_/EC_50_ > 0.007 and EC_50_ and CC_50_ in Vero CCL-81 SARS-CoV-2 cells; (2) or having been tested or considered for clinical testing in individuals with COVID-19; (3) or reported active in an in vivo SARS-CoV-2 infection model; (4) or presumed active based on mechanistic studies.

#### 2.3.1. Measurement of Half Maximal Cytotoxic Concentration (CC_50_)

The CC_50_ values of promising compounds were also measured in human PBM, Huh-7, CEM, Caco-2, and Calu-3. For the PBM, Huh-7, and CEM assays, cells were plated directly with the compound and allowed to grow under proliferative (10% FBS) conditions for 3 (Vero), 4 (Huh-7 and CEM), or 5 days (PBM). For Caco-2 and Calu-3 assay, cells were assayed under non-proliferative conditions, whereby they were grown to 90% confluence in media with 10% FBS, before replacing with fresh media containing 2% FBS and dissolved drug and incubated for 3 days. At the end of the incubation period, cells were tested for their ability to metabolize MTS (3-(4,5-dimethylthiazol-2-yl)-5-(3-carboxymethoxyphenyl)-2-(4-sulfophenyl)-2H-tetrazolium) as previously described [72]. In brief, 15 µL of MTS reagent (Promega©, Madison, WI, USA) was added per well and optical density reads were taken after 1–4 h at 490 nm absorbance on a multi-mode plate reader (Synergy, BioTek^®^, Winooski, VT, USA). After subtraction of the media-only controls, CC_50_ values were calculated using the Chou and Talalay method [79]. Drug binding in 2% FBS was expected to be negligible, so that CC_50_ measured in Calu-3 and Caco-2 cells was assumed equal to the CC_50_ of unbound drug. Unbound CC_50_ values in other cells were calculated by multiplying the measured CC_50_ values by the unbound fraction of the compound reported in human plasma, normalized to the FBS concentration in the media.

#### 2.3.2. Measurement of In Vitro Anti-SARS-CoV-2 Activity (EC_50_ and EC_90_)

The drugs were initially evaluated for anti-SARS-CoV-2 activity in Vero CCL-81 cells, followed by an evaluation in Calu-3 and Caco-2 cells, using a virus yield inhibition assay with virus quantification by qRT-PCR, as described previously [80]. Briefly, a monolayer of each cell line was prepared in a 96-well plate using media containing 10% FBS. Once the cells reached confluency, they were then treated with 2-fold serial dilutions of each compound in triplicate and infected with SARS-CoV-2 (hCoV-19/USA/WA1/2020 strain) at a multiplicity of infection (MOI) of 0.1 (Vero) and 0.01 (Calu3 and Caco2). The cells were then incubated at 37 °C for 48 h (Vero) and 72 h (Calu3 and Caco2) in the presence of 5% CO_2._ The cell infections were performed in medium containing 2% of heat-inactivated FBS. After incubation, 100 µL of the supernatant was collected into 150 µL of RLT Buffer (Qiagen^©,^ Germantown, MD, USA) for downstream RNA extraction (RNeasy 96 extraction kit, Qiagen^©^, Germantown, MD, USA) and subsequent qRT-PCR to detect the viral load using a 6-carboxyfluorescein (FAM)-labeled probe with primers against the SARS-CoV-2 non-structural protein 3 (nsp3). (SARS-CoV-2 FWD: AGA AGA TTG GTT AGA TGA TGA TAG T; SARS-CoV-2 REV:TTC CAT CTC TAA TTG AGG TTG AAC C; SARS-CoV-2 Probe: 56-FAM/TC CTC ACT GCC GTC TTG TTG ACC A/3BHQ_1).

The antiviral NAs and nucleoside base precursors, other than favipiravir, were evaluated up to 20 μM (>C_max_ observed in the clinic) [81,82,83,84]. Favipiravir (AVIGAN^TM^, Taisho Toyama Pharmaceutical Co., Ltd., Subsidiary of Fujifilm, Tokyo, Japan) was evaluated up to 300 μM, as it is administered at a considerably higher dose and has a higher C_max_ than the other NAs tested (Table 1) [85,86]. Other drugs were tested up to 100 μM. Only drugs with measurable, low EC_50_ values were retested to ensure reproducibility.

### 2.4. Pharmacokinetics (PK) and Assessment of the Relative Clinical Potential of Compounds

Peak plasma concentrations (C_max_, μM = ng/mL/MW, where MW is the molecular weight of the compound) and the unbound fraction of most drugs were obtained from the literature, or from FDA-approved package inserts. The unbound fraction of apilimod (not found in the literature) was estimated based on chemical structure, using a PK computer program, which uses a random forest machine learning method (DruMAP ver. 1.5, Mizhguchi Laboratory, Tokyo, Japan, https://drumap.nibiohn.go.jp/, Accessed 5 April 2024) [87]. Drugs that exhibited binding to plasma proteins were assumed to equilibrate rapidly between the protein-bound and unbound fractions in the plasma, and that only the unbound fraction was free to interact with cells [88]. The unbound concentration of drug in the plasma at C_max_ (unbound C_max_) was calculated by multiplying C_max_ by the unbound fraction of drug.

**Table 1:** Anti-SARS-CoV-2 activity and C_max_/EC_50_ of drugs in Calu-3, Caco-2, and Vero CCL-81 cells.

	C_max_ (μM)		Calu-3 SARS-CoV-2		Caco-2 SARS-CoV-2		Vero CCL-81 SARS-CoV-2		Therapeutic Category	Refs
Drug Typical dose	(% CV or range) {unbound}	F_un_	EC_50_/EC_90_ (μM)	Unbound C_max_/EC_50_	EC_50_/EC_90_ (μM)	Unbound C_max_/EC_50_	EC_50_/EC_90_ (μM)	unbound C_max_/EC_50_	NA	
* VV116 300 mg bid	10.6 (25%) {8.37}	---	---	34	_---_	105	---	10.5	NA	[89]
** Molnupiravir (NHC) 800 mg bid	8.99 (37%) {7.10}	1	0.4/1.8	22	1.7/12.2	5.3	0.6/1.4	4.58	NA	[17]
* Obeldesivir 350 mg bid	7.33 (32%) {5.79}	---	---	19	---	72	---	7.2	NA	[28]
GS-441524 750 mg/d	3.05 (23%) {2.41}	0.79	0.3/2.34	8.03	0.08/1.4	30	0.8/1.4	3.0	NA	[90,91]
Remdesivir 200 mg d1, 100 mg qd	3.70 (19%) {0.44}	0.12	0.2/0.7	2.2	0.02/0.1	22	3.2/4.7	0.14	NA	[10]
Nirmatrelvir 300 mg + 100 mg ritonavir bid.	6.86 (33%) {2.13}	0.31	1.8/8.8	0.4	0.1/0.3	6.8	3.7/5.6	0.21	NNAV	[92]
Nitazoxanide	1.4 (19%) {0.014}	0.01	22.8/42.9	0.015	4.4/11.1	0.078	1.4/3.9	0.25	APA	[93,94]
Ivermectin 390–470 μg/kg qd	0.30 (66%) {0.28}	0.07	6.2/9.3	0.003	4.5/8.9	0.005	2.1/4.5	0.010	APA	[71,95,96,97]
Imatinib 400 mg qd	5.17 (30.3%) {0.26}	0.05	77.9/95.8	0.003	37.9/88	0.006	4.6/14.5	0.056	ONC	[98]
Apilimod mesylate 150 mg qd	0.54 {0.043}	0.08	>100/NE	NE	85/>100	0.0005	0.7/7.4	* 0.062	ILi	[99,100]
Celecoxib 200 mg bid	1.85 (38%) {0.056}	0.03	3.0/6.3	0.019	0.7/1.2	0.08	7.2/14.6	0.008	NSAID	[101]
Zileuton 600 m qd	2.29 {0.16}	0.07	>100/NE	NE	>100	0.003	6.7/35.8	0.02	LM	[102]
Daclatasvir 60 mg qd	1.91 (13%) {0.02}	0.01	>100/NE	NE	44.7/89.4	0.0005	0.7/3.5	0.03	NNAV	[103]
Fenofibrate 200 mg qd (micronized form)	28.36 (19%) {0.28}	0.01	>100/NE	NE	>100/NE	NE	42.7/88.7	0.007	LL	[104]
Ebselen 800 mg qd	0.256 (47%) {0.013}	0.05	6.5/>10	0.002	>100/NE	NE	1.1/2.7	0.012	AO	[105]
Favipiravir	411 (45%) {193}	0.46	>300/NE	NE	256/>300	0.74	90.6/234.1	2.1	NA	[85]
Fluvoxamine (maleate) 100 mg qd	0.039 (0.02–0.06) {0.003}	0.23	>100/NE	0.005	17.9/47.5	0.0017	>100/NE	NE	SSUi	[106,107]
Honokiol not approved	NE	0.36	69.2/94.0	69.2/94	9.6/80.4	NE	20.1/84	NE	NP	[108]
Iota-Carrageenan (topical)	---	---	0.5/1.9	---	59.2/93.3	---	0.7/1.6	---	DS	
Mefenamic acid 500 mg day 1, 250 mg qid	15.83 (11–22) {1.58}	0.1	> 100/NE	NE	>100/NE	NE	87.6/>100	0.018	NSA	[109,110]

C_max_ = peak plasma concentration, F_un_ = unbound (presumed active) fraction of drugs in plasma; EC_50_ and EC_90_ are the in vitro concentrations of drug which inhibit viral replication by 50 and 90%, respectively. CC_50_ = drug concentration which inhibits cell growth by 50% (means, % CV, or ranges (when available) and mean unbound concentrations are shown). Unbound C_max_/EC_50_ ratio = C_max_ × F_un_/EC_50_. qd = once daily, bid = twice daily, tid = three times daily. NE = not estimated with existing data. Drug binding in 2% FBS was expected to be negligible, so that the EC_50_ measured in Calu-3, Caco-2 cells, and Vero cell was assumed equal to those of the unbound (presumed active) fraction of drug. * Oral administration of obeldesivir delivers GS-441524 into the plasma. VV116^TM^ delivers the deuterated form of GS-441524 into plasma, which was assumed to have similar PK as the non-deuterated form. Therefore, the C_max_ and cellular pharmacology of these drugs are reported in terms of GS-441524 (tabulated as ---). ** Oral administration of molnupiravir delivers NHC in the plasma, so the C_max_ and cellular pharmacology is reported in terms of NHC (β-D-N^4^-hydroxycytidine). The drugs are listed in descending order of the ratio of unbound drug in plasma to antiviral potency SARS-CoV-2 measured in vitro (C_max_/EC_50_) in the order: Calu-3, Caco-2, and Vero cells. NE = not estimated. Therapeutic category; AO: antioxidant; APA: anti-parasitic agent; DS: dietary supplement; FA: food additive; AH: histamine 1 or 2 blocker; IL: inhibitor of IL-12 and IL-23 production; LL: lipid-lowering drug; LM: leukotriene modulator; NA: antiviral analog of a nucleoside or nucleoside base; NNAV: non-nucleoside and non-nucleoside-base analog antiviral agent; NSAID: non-steroidal anti-inflammatory drug.

## 3. Results

Table 1 summarizes the anti-SARS-CoV-2 activities in human Calu-3 and Caco-2 cells, and in the Vero CCL 81 cells. Drugs are tabulated in descending order of their mean unbound Cmax/EC_50_ ratios in rank order of Calu-3, Caco-2, and Vero CCL-81 cells, respectively. Higher ratios were assumed to correspond with a greater in vivo potential for efficacy. This ratio is considered a lenient indicator of potential efficacy of compound, as plasma concentrations typically decline shortly after oral delivery or after the completion of IV infusion [71].

The toxicities of the drugs in Calu-3, Caco-2, Vero CCL-81, PBM, CEM, and Huh-7 cells are listed in Table 2. Drugs are listed in the same order as in Table 1.

Table 3 summarizes the anti-SARS-CoV-2 activity, cellular toxicity, and C_max_ at typical dose regimens of drugs tested only in Vero CCL-81. The following drugs were not tabulated due to a lack of antiviral activity in Vero CCL-81 cells: NAs and nucleoside-base precursors (emtricitabine, lamivudine, ribavirin, sofosbuvir, tenofovir alafenamide, tenofovir disoproxil fumarate, kinetin). Drugs which were inactive at concentrations up to 100 μM included: cetirizine (antihistamine), dexamethasone (glucocorticoid), L-ascorbic acid (antioxidant), fluvoxamine maleate (serotonin re-uptake inhibitor), metformin (used to treat diabetes), and plerixafor (used to treat malignancies).

The EC_50_, EC_90,_ and CC_50_ values for NA and nucleoside-base analogs were especially celldependent (discussed below). Most drugs with anti-SARS-CoV-2 activity had unbound EC_50_ values similar to or below the CC_50_ values in the cells used for the assay, suggesting that their antiviral activity was not due to cytotoxicity per se. Except for certain antiviral NAs, the EC_50_ values in the Vero cells were lower than in the Caco-2 or Calu-3 cells (discussed below). The NAI developed as anti-SARS-CoV-2 agents had the highest protein adjusted C_max_/EC_50_ ratios in Calu-3 (2.2 and 34) and Caco-2 cells (2.2 to 34). The SARS-CoV-2 protease inhibitor, nirmatrelvir (active component of Paxlovid^TM^), had an adjusted C_max_/EC_50_ ratio = 0.42 and 6.8 in Calu-3 and Caco-2 cells, respectively. Drugs that were ineffective in the clinic had considerably lower C_max_/EC_50_ ratios (e.g., ivermectin had C_max_/EC_50_ ratios = 0.003 and 0.005 in Calu-3 and Caco-2 cells, respectively). The remaining drugs had C_max_/EC_50_ ratios that were considerably lower than that of ivermectin in these human cell lines.

## 4. Discussion

### 4.1. Relevance of Cell Lines and SARS-CoV-2 Variants

Since the start of the COVID-19 pandemic, many studies have investigated the possible repurposing of existing medications for the treatment of this disease [42,128,129]. In the present study, initial antiviral evaluations were performed in Vero CCL-81 cells, a kidney epithelial cell line derived from the African Green monkey. Vero cells are commonly used for in vitro evaluation as they are highly permissive to SARS-CoV-2, possibly related to a defective interferon response [42]. Certain drugs, such as chloroquine and hydroxychloroquine (not tested in this study), gained emergency approval from the US FDA during the start of the pandemic, but proved ineffective [42]. Initial optimism may have been fueled by in vitro potency reports on these drugs which relied mainly on experiments performed in Vero cells [130]. SARS-CoV-2 binds the cellular receptor ACE2 and must be activated by proteolysis with either a surface-expressed protease, like TMPRSS2, or by cathepsin L in the endosome [131]. In Vero cells, SARS-CoV-2 is primarily activated by cathepsin L in acidic endosomes, where it is uncoated before being released into the cytoplasm [132]. Chloroquine and hydroxychloroquine are di-protonic weak bases which raise the pH of the endosomes, thereby inhibiting virus uncoating and replication. However, in human lung and colon cells (primary sites of infection), SARS-CoV-2 is primarily activated by TMPRSS2, which does not require lysosomal activation and is not inhibited by chloroquine [131].

SARS-CoV-2 primarily infects the lung and GI tract in individuals with COVID-19 [132]. Therefore, the drugs that demonstrated potency in Vero cells were further evaluated in Calu-3 cells, a lung epithelial cell line originally derived from a 23-year-old man with a lung sarcoma, and Caco-2 cells, a cell line derived from a 72-year-old man with colorectal cancer. Notably, the EC_50_ and CC_50_ values of the drugs differed in the various cell systems.

Data from the Calu-3 cells are prioritized over that of the Caco-2 cells in Table 1, since lung involvement is the most common serious manifestation of COVID-19, ranging from asymptomatic disease or mild pneumonia to severe disease associated with hypoxia, and critical disease associated with shock, respiratory failure, and multiorgan failure or death. However, Caco-2 data are also considered important since the GI symptoms during a COVID-19 infections can be severe [132].

The SARS-CoV-2 isolate, hCoV-19/USA-WA1/2020, NR-52281, was used in this study as it is well studied in the literature, which allows our data to be compared with other studies. However, since the virus continues to mutate, further studies may be indicated should drug-resistant strains emerge [2,3].

### 4.2. Potent Antivirals: Unbound C_max_/EC_50_ Ratios > 1 in Calu-3, Caco-2, and Vero CCL-81 Cells

Of the six drugs in this category, five are NAs, including remdesivir, an FDA-approved IV-administered prodrug of GS-441524 (used as a positive control in the in vitro assays), and orally administered drugs, GS-441524, VV116 (prodrug of a deuterated version of GS-441524), Obeldesivir (a prodrug of GS-441524), and molnupiravir (prodrug of NHC). Nirmatrelvir, the SARS-CoV-2 protease inhibitor in Paxlovid^TM^, is also included in this category.

In general, the NAs enter cells rapidly, via the equilibrative nucleoside transporters 1 and 2 (Ent 1 and 2), and undergo intracellular phosphorylation to their nucleoside 5′-triphosphate (NTP) forms, which inhibit SARS-CoV-2 RNA polymerase, composed of the nonstructural protein 12 (nsp12) and the co-factors, nsp7/nsp8, a key enzymatic system responsible for the replication of the viral RNA [133,134]. Following the administration of NAs to patients, cellular concentrations of the accumulated NTP decline slower than NAI in the plasma. Therefore, the dosing frequency of the antiviral NAs are typically related to the respective cellular stabilities (t_1/2_) of the NA-TP in the tissues susceptible to infection [135,136].

TheIV-administered remdesivir had unbound C_max_/EC_50_ ratios of 2.2, 22, and 0.14 in Calu-2, Caco-2, and Vero CCL-81 cells, respectively. Remdesivir was nontoxic in Vero CCL-81, Calu-3, and Caco-2 cells (CC_50_ > 100 μM), but PBM cells, CEM, and Huh-7 cells were found to be sensitive (CC_50_ = 3.7, 10.1 and 1.9, μM, respectively). Remdesivir is a chiral phosphoramidate protide of GS-441524 which increases the lipophilicity of the drug and enhances its ability to penetrate lipid cell membranes via diffusion [137]. Intracellular esterase-1 (CES1), cathepsin-A (CTSA), and the phosphoramidase HNT1 then convert it to GS-441524 monophosphate (MP), thereby bypassing the rate limiting initial phosphorylation to GS-441524-MP. GS-441524-MP is rapidly phosphorylated by cellular kinases to the active 5′-triphosphate form (GS-443902, GS-441524-TP), which acts as a competitive non-obligate chain terminator of RdRp during viral replication.

The differing EC_50_ and CC_50_ values between cell lines may result from elevated cellular levels of carboxylesterase 1 (CES1) and cathepsin A (CatA) in various cell lines. Although remdesivir is rapidly cleared from the plasma following IV infusion (plasma t_1/2_ ~ 1 h), it is administered once per day due to the cellular stability of GS-441524-TP (e.g., the t_1/2_ of GS-441524-TP in human blood mononuclear cells following an IV infusion over 2 h, in vivo, is about 36–43 h [138]). In contrast, the NTP of NHC has a cellular t_1/2_ of 4–5 h in human astrocytes and human bronchial tracheal epithelial cells (hBTEC), and is significantly less (0.4–1.1 h) in primary hepatocytes [139]. Oral administration of remdesivir is not feasible for treating SARS-CoV-2 lung infections, as the phosphoramidate protide is de-esterified during the first-pass metabolism by CES1, forming GS-441524, which is phosphorylated in hepatocytes before reaching the systemic circulation [140,141,142]. Studies in African Green monkeys revealed that <1% of orally administered remdesivir enters the systemic circulation intact [143]. Jubilant Pharma Ltd. reported on an oral formulation of remdesivir designed “to sidestep hepatic metabolism”. Safety and PK studies in animals and healthy human volunteers in India produced promising safety and absorption data, but no efficacy data were reported [144].

Orally administered derivatives of the parent NA GS-441524 are in various stages of development [142,145]. The EC_50_ of GS-441524 versus SARS-CoV-2 in Calu-3, Caco-2, and Vero CCL-81 cells were 0.3, 0.08, and 0.8 μM, respectively (Table 1). GS-441524 enters cells via nucleoside transporters, and the initial phosphorylation step forming GS-441524-MP is rate limiting, resulting in a lower cellular anti-SARS-CoV-2 activity for GS-441524 in comparison with remdesivir in primary lung cells and other cells [91,137,146]. Notably, orally administered GS-441524 improved survival and reduced lung inflammation and injury in a mouse model of SARS-CoV-2 infection [147]. However, the oral bioavailability of GS-441524 varies with species and is about 3% and 89% in Cynomolgus monkeys and dogs, respectively [145]. A PK and efficacy study of GS-441524 in an African Green monkey infection model of SARS-CoV-2 reported an oral bioavailability of <10% [148].

The unbound C_max_/EC_50_ ratios of IV-administered remdesivir in the Calu-3 cells were 2.2, versus 22 for the orally administered molnupiravir (Table 1). Taken in isolation, this would incorrectly imply that orally administered molnupiravir has a greater efficacy than IV-administered remdesivir. However, the EC_50_ values used in the calculation were measured in vitro after hours-long incubation, when the ratio of NAs in the medium to their respective intracellular NA-TPs were at a steady state and does not reflect differences in the plasma t_1/2_ (e.g., 0.5 h for remdesivir versus 3.3 h for NHC, and 6–7 h for GS-441524). It also does not reflect differences in the accumulation dynamics of the NA-TP, which may be 16-fold slower for GS-441524 than remdesivir [143]. Nor does it reflect differences in the cellular decay half-lives of the various NA-TPs. The combined effect(s) of these factors on the dynamics of NA-TP accumulation during treatment could be assessed, if needed, using more comprehensive pharmacometrics models [149,150].

The SARS-CoV-2 protease inhibitor, nirmatrelvir (coadministered with the Cyp-3A4 inhibitor ritonavir in Paxlovid^TM^), is also considered a potent antiviral as it has unbound C_max_/EC_50_ ratios of 0.4, 6.8, and 0.21 in the Calu-3, Caco-2, and Vero CC-L81 cells, respectively. Paxlovid administered in the early stages of infection prevented serious COVID-19 illness and hospitalization by >95% [151,152].

### 4.3. Drugs with Moderate Unbound C_max_/EC_50_ Ratios in Calu-3 and/or Caco-2, and Vero CCL-81 Cells

Nitazoxanide is a broad-spectrum thiazolide antiparasitic agent, approved for the treatment of *Cryptosporidium parvum* and *Giardia duodenalis* infections in children aged ≥ 1 year and in adults [93]. It had an unbound C_max_/EC_50_ ratio of 0.25 in Vero CCL-81 cells, suggesting a weak potential for in vivo efficacy. However, the unbound C_max_/EC_50_ ratios of nitazoxanide in the human Calu-3 and Caco-2 cells were 0.015 and 0.078, respectively (Table 1), suggestive of a low potential for in vivo efficacy. The EC_50_ in the Caco-2 cells (4.4 μM) was less than 2-fold lower than the CC_50_ versus the Caco-2, Vero, and PBM cells (Table 2), suggestive of a potential toxicity at effective antiviral concentrations. Nitazoxanide is rapidly metabolized to its active metabolite, tizoxanide, and displays in vitro antiviral activity against a range of viruses, including the influenza viruses, hepatitis B and C viruses, norovirus, rotavirus, Ebola virus, Middle East respiratory syndrome coronavirus (MERS-CoV), and SARS-CoV-2. Nitazoxanide inhibits host enzymes, which impairs the post-translational processing of viral proteins. It also inhibits pro-inflammatory cytokines.

Due to its reported in vitro inhibition of SARS-CoV-2 replication and favorable PK of nitazoxanide, the COVER randomized trial, conducted in Johannesburg, South Africa, evaluated whether nitazoxanide or a combination of sofosbuvir plus daclatasvir could lower the risk of SARS-CoV-2 infection in 828 healthcare workers and others at high risk of infection. Neither nitazoxanide nor sofosbuvir plus daclatasvir significantly prevented SARS-CoV-2 infection in healthcare workers and others at high risk of infection [153]. The COVID-19 Treatment Guidelines Panel of the NIH recommends against the use of nitazoxanide for the treatment of COVID-19, except in a clinical trial [154]. Daclatasvir, an inhibitor of the HCV nonstructural protein, NS5A, had unbound C_max_/EC_50_ ratios of 0.03 and 0.0005 in Vero CCL-81 and Caco-2 cells, respectively. However, it was inactive in the Calu-3 cells (EC_50_ > 100 μM). In the same clinical trial, COVER, a combination of sofosbuvir with daclatasvir did not demonstrate protect against infection compared to a no-intervention control arm of the study [155].

Ivermectin had unbound C_max_/EC_50_ ratios equal to 0.003, 0.005, and 0.01 in Calu-3, Caco-2, and Vero CCL-81 cells, respectively, suggestive of a marginal antiviral effect at clinical doses. The unbound C_max_/EC_50_ ratio in the Vero CCL-81 cells is similar to the ratio reported by Peña-Silva et al. in Vero E6 cells. Their report concluded that ivermectin is unlikely to be clinically effective for the prevention or treatment of COVID-19 [72]. A multicenter open-label, randomized, controlled adaptive platform pharmacometrics trial of high-dose ivermectin in individuals with early COVID-19 (PLATCOV) showed that ivermectin does not increase the rate of SARS-CoV-2 clearance from the oral compartment [156]. The current NIH COVID-19 Treatment Guidelines do not recommend the use of ivermectin for the prevention or treatment of COVID-19 [157].

Favipiravir was inactive in Calu-3 cells (EC_50_ > 300 µM) but had activity in Caco-2 at high concentrations (EC_50_ = 256 µM, unbound C_max_/EC_50_ = 0.74) and in Vero CCL-81 cells (EC_50_ = 90.6 µM, unbound C_max_/EC_50_ = 2.1). It is a pyrazine carboxamide nucleosidase precursor, which is metabolized intracellularly to its active form, favipiravir-ribofuranosyl-5′-triphosphate, and may inhibit viral replication by inducing lethal mutations in the viral genome, in a mechanism similar to NHC [158]. Favipiravir (AVIGAN, T705 tablets, Taisho Toyama Pharmaceutical Co., Ltd, a Subsidiary of Fujifilm, Tokyo, Japan) was approved in Japan in 2014 for stockpiling for pandemic preparedness only, but not yet for the treatment of seasonal influenza. Moreover, it was marketed in China as a second-line treatment of novel or reemerging influenza outbreaks. However, favipiravir has poor antiviral activity in primary human respiratory cells, in agreement with the lack of activity observed in the human Calu-3 cells (Table 1) [157]. The drug is administered at high doses to treat influenza, as it has low to moderate antiviral efficacy at lower dosages [86]. Four separate mammalian species administered with favipiravir at doses equivalent to human regimens demonstrated delayed development or embryonic death in the first trimester, so this drug is not recommended for use in pregnant women or children [158]. A systematic review and meta-analysis of 12 clinical trials, involving 1636 patients with moderate to severe COVID-19, did not reveal significant differences in fatality and the requirement of mechanical ventilation between individuals treated with favipiravir and with standard of care [159]. 

Imatinib, a small molecule inhibitor targeting multiple tyrosine kinases, used to treat chronic myelogenous leukemia (CML) and acute lymphocytic leukemia (ALL), was weakly active in the Calu-3 (EC_50_ = 77.9 µM) and Caco-2 (37.9 µM) cells and more active in Vero CCL-81 cells (4.6 µM), with unbound C_max_/EC_50_ ratios of 0.003, 0.006, and 0.056, respectively [64,99]. A randomized, double-blind, placebo-controlled clinical trial conducted in 385 adult patients with severe COVID-19 infections did not meet its primary outcome of reducing the time needed to remain on a ventilator and the need for supplemental oxygen for more than 48 consecutive hours in patients requiring supplemental oxygen [64].

Celecoxib, a COX-2 enzyme inhibitor used for the treatment of rheumatoid arthritis, had unbound C_max_/EC_50_ ratios of 0.019, 0.08, and 0.008 in the Calu-3, Caco-2, and Vero CCL-81 cells, respectively, suggesting weak antiviral activity. However, since pro-inflammatory mediators, such as COX-2, p38 MAPK, IL-1b, IL-6, and TGF-β, play pivotal roles in coronavirus-related cell death, cytokine storm, and pulmonary interstitial fibrosis, blocking these mediators of inflammation with celecoxib could be beneficial in treating individuals with mild to moderate infections [43,44].

Apilimod is a specific inhibitor of PIKfyve kinase, a lipid enzyme which is also involved in the transport of filoviruses, including the Ebola virus and Marburg virus, into cells, and was originally developed to treat rheumatoid arthritis and Crohn’s disease [160,161]. Apilimod had unbound C_max_/EC_50_ ratios of 0.062 and 0.0005 in the Vero CCL-81 and Caco-2 cells, respectively, but was inactive in the Calu-3 cells. Apilimod inhibited SARS-CoV-2 replication in human pneumocyte-like cells derived from induced pluripotent stem cells, and in a primary human lung explant model, but worsened disease in a COVID-19 murine model [144,162,163]. The binding of apilimod in plasma was not previously reported; however, a computer analysis based on chemical structure (Section 2.4) estimated an unbound fraction of 0.062, which suggests that the discordance between in vitro potency and in vivo efficacy may result from binding to plasma proteins in vivo. AI Therapeutics sponsored a randomized, double-blind, placebo-controlled clinical trial in individuals to test the safety, tolerability, and efficacy of orally administered apilimod for the prevention of COVID-19 progression [164]. The trial was completed, but as of now the results have not been published. Baranov et al. raised a concern that since apilimod might prevent viral invasion by inhibiting host cell proteases, the same proteases that are critical for the antigen presentation leading to T-cell activation, and thus this could dampen antibody production. In addition, there is evidence from both in vitro studies and the clinic that apilimod blocks antiviral immune responses [165].

Zileuton had weak predicted efficacy in the Vero (unbound C_max_/EC_50_ = 0.02) and Caco-2 cells (C_max_/EC_50_ = 0.003) and was inactive in the Calu-3 cells (EC_50_ > 100 µM). Although leukotriene inhibitors are unlikely to inhibit SARS-CoV-2 replication, they may inhibit SARS-CoV-2-related inflammation [166]. A retrospective study reported that leukotriene inhibitors in combination with dexamethasone provided a mortality benefit in hospitalized patients with COVID-19 presenting with an oxygen saturation of below 50%. A cohort of patients in that study that received leukotriene inhibitors without dexamethasone had lower markers of inflammation and reduced cytokine storm [167].

Ebselen had an unbound C_max_/EC_50_ ratio of 0.012 in the Vero CCL-81 cells, 0.002 in the Calu-3 cells, and was inactive in the Caco-2 cells (EC_50_ > 100 µM). Ebselen is a synthetic organoselenium compound which mimics the action of glutathione peroxidase and peroxiredoxin enzymes [168]. Ebselen forms selenosulfide bonds with thiols, which results in antiviral, antibacterial, and anti-inflammatory effects [105,169]. It has been proposed that the main protease (M^pro^) of SARS-CoV-2 is a potential drug target of ebselen [55]. A clinical trial has been enrolled with orally administered ebselen (SPI-1005), sponsored by Sound Pharmaceuticals Inc. [170].

### 4.4. Drugs with Measurable EC_50_ in Caco-2 Cells and EC_50_ > 100 μM in Calu-3 and Vero CCL-81 Cells

Fluvoxamine had an EC_50_ of >100 in the Vero CCL-81 and Calu-3 cells, and EC_50_ of 17.9 μM (unbound C_max_/EC_50_ ratio = 0.0005) in the Caco-2 cells. Fluvoxamine is a selective serotonin reuptake inhibitor [171] approved by the FDA for the treatment of obsessive-compulsive disorder and is widely used for other conditions, including depression, but is not approved for the treatment of any infection. Fluoxetine, a related drug, was reported to block SARS-CoV-2 replication efficiently ex vivo in human lung tissue [172]. An observational multicenter retrospective study with fluoxetine, including 7230 adults hospitalized for COVID-19, reported a significant association between antidepressant use and the reduced risk of intubation or death (*p* < 0.001), suggesting that anti-depressant drug use could be associated with lower risk of death or intubation in patients hospitalized for COVID-19 [171]. A retrospective cohort study using a database containing 83,584 patients with COVID-19 in 87 healthcare centers across the US, which included 3401 adult patients with COVID-19 who were prescribed either fluoxetine hydrochloride or fluvoxamine maleate (SSRIs), also reported a significant association between fluvoxamine or fluoxetine use and the reduced risk of death compared to a matched control, which was not observed in outpatients administered with other SSRIs [173]. Guo et al. performed a fixed effects and sensitivity meta-analysis on the combined data (2196 patients) from three clinical trials (STOP COVID 1 and 2, and the TOGETHER Trial) to assess the use of fluvoxamine during the early stage of a COVID-19 infection for reducing the risk of hospitalization, relative to the control group. The study concluded that although patients receiving fluvoxamine were 31% less likely to exhibit clinical deterioration or hospitalization compared with the placebo, more evidence from future trials is warranted to support this finding. The COVID-19 Treatment Guidelines Panel stated that there is presently insufficient evidence to recommend either for or against the use of fluvoxamine for the treatment of COVID-19 [174].

### 4.5. Drugs with Measurable Unbound C_max_/EC_50_ Ratios in Vero Cells Only

Fenofibrate is used to treat dyslipidemia [175]. Its lipid-modifying effects are mediated by the activation of peroxisome proliferator-activated receptor-α. It also reduces the levels of fibrinogen, C-reactive protein, and various pro-inflammatory markers [176]. The fenofibric acid metabolite was shown to destabilize the receptor binding domain of SARS-CoV-2 and to reduce virus replication in the Vero cells (EC_50_ = 7 μM) [177]. In our assays, fenofibrate was weakly active in the Vero CCL-81 cells (EC_50_ = 42.7 μM, C_max_/EC_50_ ratio = 0.007), and was inactive in the Calu-3 and Caco-2 cells. A multinational double-blinded trial was conducted in 701 inpatients and nonhospitalized patients with COVID-19 within 14 days of symptom onset, who were randomized to receive 145 mg of an oral fenofibrate formulation qd (*n* = 351) versus placebo (*n* = 350) for 10 days (NCT04517396) [67]. No significant differences were observed between the arms of the study using a “symptoms severity score” metric, which considered time to death, duration of mechanical ventilation, oxygenation, hospitalization, and symptom severity (primary endpoint). Also, no differences were observed in all-cause death across the arms’ secondary and exploratory endpoints.

Mefenamic acid had an unbound C_max_/EC_50_ ratio of 0.018 in the Vero CCL-81 cells and was inactive in the Caco-2 and Calu-3 cells (EC_50_ > 100 µM). A randomized double-blind placebo-controlled trial in 38 ambulatory individuals with COVID-19 reported that patients receiving mefenamic acid with standard medical care had an about 16-fold higher probability of achieving patient-acceptable symptoms on day 8 of treatment, compared with those receiving a placebo plus standard medical care, and had reduced symptomatology [178]. Since mefenamic acid is a non-steroidal anti-inflammatory agent, it was unclear whether the clinical benefit was due to modulation of inflammation or other mechanisms.

### 4.6. Drugs with EC_50_ > 100 μM in Calu-3, Caco-2, and Vero CCL-81 Cells

Kinetin (MB-905, *N*^6^-furfurylaminopurine) previously proved ineffective for the treatment of patients with familial dysautonomia [84,179]. Kinetin was reported to inhibit SARS-CoV-2 replication in vitro at sub-micromolar concentrations in human hepatic and pulmonary cell lines, and to reduce viral replication, IL-6, and TNF levels in infected monocytes [180]. Kinetin riboside 5′-TP) inhibits the SARS-CoV-2 RNA polymerase with an IC_50_ three-fold higher than that of GS-443902-TP (the active cellular metabolite of remdesivir). Kinetin was reported to produce a similar error prone catastrophic replication of the exonuclease of the viral RdRp as molnupiravir, but had negative Ames and micronucleus tests, suggesting a low potential for mutagenesis. The kinetics of cellular accumulation and stability of kinetin riboside 5′-TP were not reported in that study. Kinetin had a 50% oral bioavailability and exhibited satisfactory plasma PK in mice and rats and was ~80% bound to plasma proteins SARS-CoV-2-infected transgenic mice expressing human ACE2 treated with an oral dose of kinetin (140 mg/kg per day) exhibited a decrease in viral replication of the gamma variant, and reduced lung necrosis, hemorrhage and inflammation, and increased survival at plasma concentrations lower than those achieved and shown to be safe in clinical trials of kinetin in patients with familial dysautonomia [179,180]. Paradoxically, in our assays, kinetin did not inhibit SARS-CoV-2 and was not cytotoxic up to 100 µM. However, significant differences in the experimental design and use of the gamma variant by Souza et al. (we employed the Washington strain) could potentially explain these differences.

Metformin, a biguanide molecule, is the main first-line drug for the treatment of type 2 diabetes, particularly in overweight individuals [181]. Metformin was reported to inhibit SARS-CoV-2 replication in cell culture and in human lung ex vivo at physiologically relevant concentrations [182,183,184]. However, in our assays, we did not observe an EC_50_ or cytotoxicity at concentrations up to 100 μM (10-fold higher than the plasma C_max_ (bound + unbound) = 10.2 μM) [185]. Retrospective studies reported an association between metformin use and less severe COVID-19 in patients already administering metformin [186,187,188]. The COVID-OUT trial was a Phase 3, randomized, placebo-controlled trial conducted in the United States from 21 May 2021 to 28 January 2022, which tested the effectiveness of metformin, ivermectin, and fluvoxamine in preventing serious SARS-CoV-2 infection in non-hospitalized adults. The study found no reduction in hypoxemia, emergency room visits, hospitalization, or death associated with any of the three drugs [189]. A secondary analysis of the data suggested that metformin may reduce a composite of emergency room visit, hospitalization, or death in overweight or obese individuals with COVID-19, and that further studies with metformin may be warranted in a similar population. The trial was lengthened to study the effects of these drugs on long COVID, a heterogeneous group, ranging from a single symptom to serious multi-organ involvement, and from mild and short-lived to chronically debilitating [190,191]. The study concluded that outpatient treatment with metformin reduced the incidence of long COVID by about 41%. The absolute reduction in long COVID incidence was 4.1%, compared with the placebo, suggesting only a marginal benefit [192]. The exact pathophysiology of long COVID is unknown, but it is likely to be multifactorial, including the inflammatory cascade during an acute infection and persistent viral replication [193].

### 4.7. Drugs with EC_50_ < 100 μM in Vero-CCL-81, Calu-3, and Caco-2 Cells but without Published Human PK Data

Honokiol is a natural polyphenolic compound extracted from the bark and leaves of the magnolia grandiflora, and is used in traditional medicine for treating a variety of ailments (e.g., malignancies, neurologic diseases, muscle spasms, depression, thromboses, microbial infections, and others) [194]. Tanikawa et al. reported that honokiol partially inhibited the furin-like enzymes responsible for cleaving a motif on the S1/S2 boundary of the spike (S) protein of SARS-CoV-2, and inhibited SARS-CoV-2 infectivity in Vero E6 cells with EC_50_ = 13 μM, and CC_50_ = 54 μM [195]. They concluded that honokiol and crude drugs which contain honokiol may benefit individuals with COVID-19. In our assays, the EC_50_ and unbound CC_50_ values of honokiol were as follows: 20.1 versus 37.8 μM in the Vero CCL-81 cells; 69.2 versus 27.5 μM in the Calu-3 cells; and 9.6 versus 18.4 μM in the Caco-2. The unbound CC_50_ values in human PBM, CEM and Huh7 cells were 26.5, 26.7, and 12.4 μM, respectively, indicative of toxicity at concentrations similar to those needed to inhibit SARS-CoV-2 replication. The PK of honokiol in humans remains unpublished. However, studies in rats and mice indicate that oral absorption is limited by poor solubility, intestinal metabolism during absorption, and first-pass hepatic metabolism. Magnolol, a similar compound (2,2-diol versus 2,4-diol) extracted from the bark and leaves of the magnolia grandiflora, has a 4% oral bioavailability in rats [196]. These data suggest that honokiol is unlikely to benefit individuals with COVID-19.

### 4.8. Compounds for Intranasal Administration to Protect against SARS-CoV-2 Infection

Carrageenans are naturally occurring, linear, sulfated polysaccharides extracted from red edible seaweeds, and they are widely used in the food industry as gelling, thickening, and stabilizing agents [197]. Iota-carrageenan was previously reported to inhibit SARS-CoV-2 replication in Vero cells [198]. In our assays, iota-carrageenan had an EC_50_ of 0.7, 0.5, and 59.2 μM in the Vero CCL-81, Calu-3, and Caco-2 cells, respectively. It had a CC_50_ of 95.9 μM in the Vero CCL-81 cells and >100 μM in the other cell systems tested. Previous animal experiments, which included repeated dose, local tolerance, and toxicity studies with intranasally applied 0.12% iota-carrageenan for 7 or 28 days in New Zealand White rabbits, as well as nebulized 0.12% iota-carrageenan administered to F344 rats for 7 days, revealed no penetration of iota-carrageenan across the nasal mucosa into the blood stream. The data do not provide any evidence for local intolerance or toxicity when carrageenan is applied intranasally or by inhalation. No signs for immunogenicity or immunotoxicity have been observed in the in vivo studies [199]. However, a pilot study of a topical nasal spray containing iota-carrageenan, in 300 hospital personnel dedicated to caring for patients with COVID-19 disease, demonstrated the following relative risk reduction: 79.8% (95% CI 5.3 to 95.4; *p* = 0.03). However, the absolute risk reduction was 4% (95% CI 0.6 to 7.4), suggesting a marginal benefit in this population [200].

## 5. Conclusions

There is a need for additional safe, effective, and inexpensive medications for the treatment or prevention of SARS-CoV-2 infection, and drug repurposing may be an option to identify them. We evaluated a wide range of drugs which were considered for repurposing. However, drugs that were not specifically developed as SARS-CoV-2 antiviral agents had the marginal ability to inhibit the replication of this virus at clinically relevant concentrations. Vero cells are highly permissive to this virus and thus widely used in the laboratory for antiviral drug evaluation. However, the data presented herein demonstrated that the potency and toxicities of some drugs, especially NA inhibitors were cell-system dependent. This was not unexpected since different cell types may have unequal distributions of drug metabolizing enzymes and membrane transporters [201], and certain antiviral agents (e.g., remdesivir and nirmatrelvir) are substrates of the Cytochrome P450 enzymes, P-glycoprotein, and other cell membrane transporters [202,203]. Also, NA phosphorylation may be cell-dependent [138,139]. It is informative to relate in vitro potency to reported in vivo plasma concentrations. The underlying assumption being that the concentration of the active (presumed unbound) form of the direct-acting antivirals (or the intracellular NTP) remains above the EC_50_ at the infection site for a sufficient fraction of the dose interval to inhibit viral replication. Many drugs that proved ineffective in the COVID-19 clinical trials exhibited an unbound peak plasma concentration (unbound C_max_) considerably less than the EC_50_ in the Calu-3 and Caco-2 lung-derived cells. Antiviral agents effective in controlled clinical trials had unbound C_max_/EC_50_ ≥ 6.8 in Calu-3 or Caco-2 cells. More comprehensive PK analysis and modeling is warranted for the drugs exhibiting higher unbound C_max_/EC_50_ ratios to assess their potential duration of efficacy. This could be achieved by superimposing a plot of simulated drug concentrations versus time profiles in plasma, generated using a PK model, with EC_50_ values in the physiologically relevant cells normalized for protein binding [204]. PK models may be expanded to include in vitro derived phosphorylation kinetics of NA (using a relevant cell line) to generate NTP versus time profiles, versus dose and time. The NTP concentration profiles can, in turn, be compared in vitro with the EC_50_, estimating the NTP concentration versus that of the viral polymerase [151,152]. Since SARS-CoV-2 infects other more shielded tissues (e.g., the central nervous system), drug exposures in other, more shielded organ sites (e.g., from animal studies) should be also considered [205].

The literature summary presented for many drugs suggests that they could be considered as possible adjunct therapies in combination with drugs known to inhibit SARS-CoV-2 replication to treat COVID-19-related symptoms in mild to moderate infections. Karim and Devranim commented in a recent editorial concerning the failure of the COVID-OUT trial on the use of oral metformin, ivermectin, or fluvoxamine to protect individuals with mild to moderate COVID-19 from severe disease (above) [206]. They concluded the following: “With respect to clinical decisions about COVID-19 treatment, some drug choices, especially those that have negative WHO recommendations, are clearly wrong. In keeping with evidence-based medical practice, patients with COVID-19 must be treated with efficacious medications; they deserve nothing less”. Similarly, an editorial by Dr. Abrescia, concerning a study which showed a lack of association between antiretroviral use and the acquisition or severe outcomes of SARS-CoV-2 infection in people with HIV in the Netherlands, recommended the following: “If SARS-CoV-2 infection does take place, drugs proven to be effective against SARS-CoV-2 (remdesivir or nirmatrelvir/ritonavir) should be started within five days of the onset of symptoms” [207,208]. Our calculations of the unbound C_max_/EC_50_ ratios and EC_50_ measurements in the cell systems relevant to in vivo SARS-CoV-2 infection lends further support to that conclusion. Many drugs may have potential in alleviating the symptoms of COVID-19 and/or in improving the outcomes of SARS-CoV-2 infection, despite having minimal or marginal direct antiviral effects. These should be tested as adjuncts during therapy with potent antiviral agents.

## Figures and Tables

**Table 2:** Toxicities of the drugs in the Calu-3, Caco-2, Vero CCL-81, PBM, CEM, and Huh-7 cells.

	Calu-3	Caco-2	Vero	PBM	CEM	Huh7
Drug	CC_50_ (μM)	CC_50_ (μM) {Unbound}	CC_50_ (μM) {Unbound}	CC_50_ (μM) {Unbound}	CC_50_ (μM) {Unbound}	CC_50_ (μM) {Unbound}
* NHC	>100	>100	18.8 {18.8}	59.8 {59.8}	2.4 {2.4}	59.1 {59.1}
GS-441524	>100	>100	>100	>100	>100	>100
Remdesivir	>100	>100	>100	4.5 {3.7}	11 {10.1}	2.1 {1.9}
Nirmatrelvir	>100	>100	>100	>100	>100	>100
Nitazoxanide	49.8	7.2	8.7 {7.8}	8.3 {6.7}	15.5 {14}	54.3 {48.9}
Ivermectin	17.5	5.8	35.2 {31.9}	29.2 {23.8}	12 {9.5}	5.6 {5.1}
Imatinib	90	66	12.3 {11.1}	13.9 {11.3}	14.1 {12.8}	11.9 {10.8}
Apilimod mesylate	>100	>100	>100	>100	68.5 {62.2}	60.3 {54.8}
Celecoxib	70.5	59.1	50 {45.2}	>100	77.1 {69.6}	31.1 {28.1}}
Zileuton	46.7	>100	>100	>100	>100	>100
Daclatasvir	>100	67.6	50.6 {45.6}	36.4 {29.2}	26.2 {23.6}	53.6 {48.3}
Fenofibrate	≥100	>100	>100	38.9 {31.2}	28.5	>100
Ebselen	>100	>100	22.4 {20.3}	93.2 {75.5}	46.7 {42.3}	84.3 {76.3}
Favipiravir	>300	>300	>100	>100	>100	>100
Fluvoxamine (maleate)	>100	>100	35.5 {32.8}	35.4 {29.9}	20.0 {18.5}	36.7 {33.9}
Honokiol	27.5	18.4	40.4 {37.8}	30.4 {26.5}	28.6 {26.8}	13.2 {12.4}
Iota-Carrageenan	>100	>100	>100	>100	95.9 {NE}	>100
Mefenamic acid	>100	>100	>100	>100	>100	>100

The unbound CC_50_ values were calculated by multiplying the measured CC_50_ values by the unbound fraction of drug reported in human plasma normalized to the FBS concentration in the media. * NHC (β-D-N4-hydroxycytidine).

**Table 3:** The anti-SARS-CoV-2 activity, toxicity, and typical C_max_ of drugs tested only in Vero CCL-81 cells.

	Vero CCL-81 Cells		C_max_ (μM)				
Drug Typical Dose	EC_50_/EC_90_ (μM)	CC_50_ (μM)	(% CV or Range) {Unbound}	F_un_	Unbound C_max_/EC_50_	Therapeutic Category	Refs
Doxycycline 100 mg bid then 100 mg/d	69.3/102	≥100	3.67 (50%) {0.55}	0.15	0.008	ABA	[111]
Clofazimine 200 mg/d	0.5/3	34.3	1.67 (2.14–2.60) {0.002}	0.001	0.003	ABA	[112]
Famotidine 40 mg bid	160/NE	>100	0.2 (29%) {0.16}	0.8	0.001	AH	[113]
Chlorpheniramine maleate 8 mg q 4–6 h	31/104	>100	0.027 (41%) {0.008}	0.28	0.0002	AH	[114]
Lotilaner 20 mg/kg, once (dog or cat)	1.9/4.9	44.3	6.72 (25%) {0.007}	0.001	0.0035	APA	[115]
Ciclesonide Inhale 80 mg bid	4.1/7.8	33.9	0.001 {0.0000/1}	0.01	1.9 × 10^−6^	GC	[116]
Montelukast sodium 10 mg/d	4.9/7.2	35.9	0.58 (0.3–0.90) {0.009}	0.01	0.002	LM	[117]
Bromhexine 16 mg bid	0.8/6.2	>100	0.093 (24% {0.005})	0.05	0.006	ML	[118]
Elbasivir 50 mg/d	3.4/12.5	>100	0.26 (47%) {0.003}	0.01	0.001	NNAV	[119]
Amantadine 129 mg bid	138/288	>100	2.17 (9%) {0.002}	0.001	0.0002	NNAV	[120]
Velpatasvir 100 mg/d	19.1/79.6	>100	0.29 (54%) {0.001}	0.005	7.8 × 10^−5^	NNAV	[81]
GC376 10 mg/kg (in cats)	0.2/0.6	>100	1.7 {NE}	NE	NE	NNAV	[121]
Cannabinol cigarette (0.79 g) with 6.8% TH	2.7/8.7	37.3	0.032 (0.012–0.051) {0.013}	0.1	0.0011	NP	[122]
Melatonin 2–6 mg/d	22/NE	>100	0.0215 (0.009–0.034) {0.005}	0.22	0.0002	NP	[123]
Diclofenac sodium 50 mg tid	97.6/141	>100	2.41 {0.012}	0.005	0.0001	NSAID	[124,125]
Flavopiridol 30 mg/m^2^ + 60 mg/m^2^ infusion	0.1/1.1	0.031	1.92 (55.7%) {0.096}	0.05	0.99	ONC	[126]
Acalabrutinib 100 mg bid	11.1/70.9	>100	0.69 {0.017}	0.025	0.002	ONC	[127]

F_un_ = 1 – (fraction bound reported in the literature). F_un_ of apilimod was computed in silico, and that of lotilaner was approximated as 0.01, as it was described as highly bound. Within each therapeutic category, compounds are listed in descending order of adjusted Cmax/EC50 in Vero CCL-81 cells. qd = once daily, bid = twice daily, tid = three times daily. NE = not estimated. Therapeutic category: ABA: antibacterial antibiotic; AF: antifungal; AH: histamine 1 or 2 blocker; AP: anti-parasitic agent; GC: glucocorticoid; APA: anti-parasitic agent; NP: natural product; ML: mucolytic; NSAID: non-steroidal anti-inflammatory drug; ONC: oncology drug; NNAV: non-nucleoside and non-nucleoside-base analog antiviral drug.

## Data Availability

All pertinent data are reported in the manuscript.

## Abbreviations

Bid	twice per day
C_max_	peak concentration of drug observed in plasma
CC_50_	drug concentration that inhibits cell division by 50% in vitro
DMEM	Dulbecco’s Modified Eagle Medium
EC_50_/EC_90_	median/90th percentile effective antiviral concentration measured in cell culture
FBS	fetal bovine serum
FDA	US Federal Drug administration
F_un_	unbound fraction of drug in plasma
H	Hour
LC-MS-MS	chemical analysis which couples liquid chromatography with mass spectrometry
MEM	Minimum Essential Media
MOI	multiplicity of infection (MOI)
MTS	3-(4,5-Dimethylthiazol-2-yl)-5-(3-carboxymethoxyphenyl)-2-(4-sulfophenyl)-2H-tetrazolium
NA	nucleoside analog
NE	not estimated
NIH	US National Institutes of Health
PD	Pharmacodynamics
PK	pharmacokinetics
Qd	once per day
t_1/2_	elimination half-life
Tid	three times per day
qRT-PCR	quantitative real-time PCR
RPMI	Roswell Park Memorial Institute 1640 Media
unbound C_max_/EC_50_	ratio of C_max_/EC_50_ normalized for protein binding.
